# Rapid clinical improvement with upadacitinib in genetically confirmed Hailey-Hailey disease after adalimumab failure^؆^

**DOI:** 10.1016/j.abd.2026.501445

**Published:** 2026-08-07

**Authors:** Burak Akşan, Emre Burakhan Akay, Elif Kundakcı Akay

**Affiliations:** a Department of Dermatology, Faculty of Medicine, Giresun University, Giresun, Turkey; b Department of Dermatology, Giresun University Training and Research Hospital, Giresun, Turkey

Dear Editor,

Hailey-Hailey disease (HHD) is an autosomal dominant genodermatosis caused by pathogenic variants in *ATP2C1*, leading to impaired keratinocyte adhesion and recurrent erosive plaques in intertriginous areas.^1^ Flares are often triggered by sweating, friction, and secondary infection; moreover, long-term disease control remains difficult in a subset of patients despite conventional therapies.^1^, ^2^ Although topical corticosteroids and antimicrobial measures are commonly used, refractory disease frequently necessitates systemic approaches, yet the supporting evidence is largely limited to small series and case reports.^2^, ^3^ Selective JAK1 inhibitors have shown clinical benefit in recent case reports.^4^, ^5^, ^6^, ^7^, ^8^ Here, we report a genetically confirmed, severe case of HHD with documented failure of prolonged weekly adalimumab therapy and rapid clinical improvement following upadacitinib.

A 34-year-old man presented with a 5-year history of recurrent, painful, exudative erosions involving both axillae, the inguinal folds, and the genital region. Histopathological examination revealed intraepidermal cleavage with prominent suprabasal acantholysis. The acantholytic keratinocytes displayed the characteristic “dilapidated brick wall” appearance, consistent with Hailey-Hailey disease. Direct immunofluorescence was negative. Genetic testing identified a heterozygous *ATP2C1* splice-site variant (c.1001+1G>A), confirming the diagnosis. He also described frequent flares triggered by heat and sweating, with a major impact on daily activities due to pain and maceration.

The patient had previously received multiple treatments with limited benefit, including topical corticosteroids and antiseptics, systemic acitretin, methotrexate, and cyclosporine. Due to persistent, extensive intertriginous involvement, the patient had also completed an 11-month course of adalimumab administered at a weekly maintenance dose of 40 mg. However, this produced no sustained benefit and was discontinued two months before our evaluation.

Cutaneous examination demonstrated active erosive axillary plaques with maceration (Fig. 1A). In addition, erythematous, macerated plaques with superficial erosions and fissures affected the inguinal folds and genital region (Fig. 1B). Given the refractory course, off-label upadacitinib 15 mg once daily was initiated. Prior to initiation, baseline laboratory screenings, including complete blood count, hepatic and renal panels, and viral/tuberculosis screening, were unremarkable. We also recommended a simple friction-reducing supportive regimen, including regular gentle cleansing, careful drying, and zinc oxide barrier paste to intertriginous areas.

**Figure 1:**
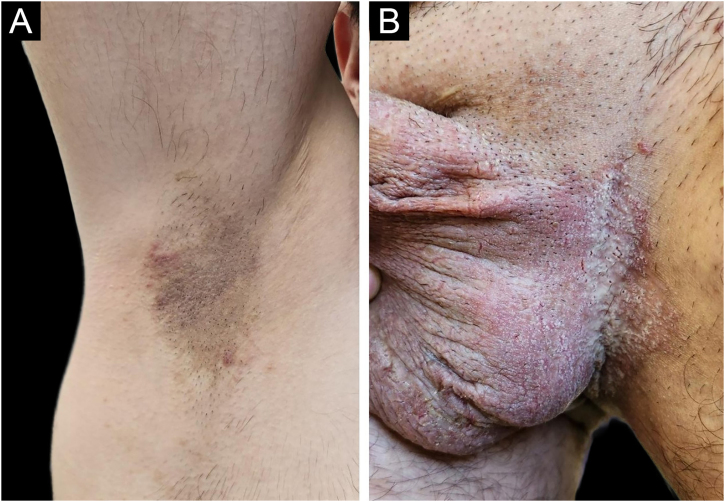
Baseline clinical findings. (A) Active erosive axillary plaque with maceration. (B) Severe inguinal and genital involvement characterized by erythema, maceration, superficial erosions, and fissuring prior to initiation of upadacitinib therapy.

The patient demonstrated a rapid and clinically meaningful response. Within the first two weeks of treatment, he reported a substantial reduction in pain and exudation. At the 4-week follow-up, axillary involvement improved markedly, with near-complete resolution of erosions and residual post-inflammatory hyperpigmentation (Fig. 2A). The inguinal and genital regions also showed marked improvement, with substantial re-epithelialization and residual mild erythema (Fig. 2B). Sustained clinical response was maintained at the 12-week follow-up, with no recurrence of erosions or fissuring. Throughout the 12-week treatment period, routine laboratory monitoring remained stable, and the patient reported no adverse events.

**Figure 2:**
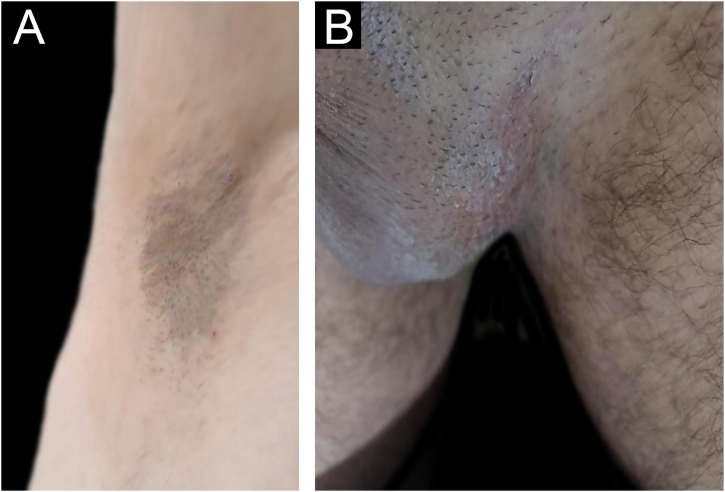
Clinical response at week-4 following initiation of upadacitinib 15 mg once daily with adjunctive zinc oxide barrier care. (A) Marked improvement of the axillary lesion, with near-complete resolution of erosions and residual post-inflammatory hyperpigmentation. (B) Marked improvement of the inguinal and genital lesions, with substantial re-epithelialization and residual mild erythema.

This case adds to the emerging evidence supporting JAK1 inhibition in HHD by documenting rapid clinical improvement after failure of a prolonged weekly TNF-α inhibitor regimen.^3^ Reported responses to biologic therapy in HHD appear heterogeneous, with TNF-α inhibitors showing variable efficacy across published cases.^3^ In one report, disease control on upadacitinib 15 mg once daily required dose adjustment and adjunct topical ruxolitinib to regain response.^6^ Abrocitinib has also been reported to improve refractory HHD, supporting the concept that JAK1-selective inhibition may be effective across agents.^7^, ^8^ Our observation is notable for marked re-epithelialization by week-4 on upadacitinib 15 mg once daily, with sustained clinical response and no reported adverse events through week-12. In summary, upadacitinib may represent a therapeutic option in carefully selected patients with refractory HHD after failure of conventional systemic agents and prior biologic therapy. However, longer follow-up is needed to evaluate its long-term efficacy and durability of response.

## ORCID ID

Elif Kundakcı Akay: 0009-0002-5959-9583

## Research data availability

Does not apply.

## Financial support

None declared.

## Authors’ contributions

Burak Akşan: Approval of the final version of the manuscript; data collection, analysis and interpretation; effective participation in research orientation; manuscript critical review; preparation and writing of the manuscript; study conception and planning.

Emre Burakhan Akay: Approval of the final version of the manuscript; data collection, analysis and interpretation; manuscript critical review; preparation and writing of the manuscript; study conception and planning.

Elif Kundakcı Akay: Approval of the final version of the manuscript; data collection, analysis and interpretation; manuscript critical review; preparation and writing of the manuscript.

## Conflicts of interest

None declared.
